# Progress test utopia

**DOI:** 10.1007/s40037-018-0413-1

**Published:** 2018-03-09

**Authors:** Cees van der Vleuten, Adrian Freeman, Carlos Fernando Collares

**Affiliations:** 1 Maastricht University, Maastrich, The Netherlands; 2 University of Exeter, Exeter, UK

**Keywords:** Progress testing, Assessment feedback, School collaboration

## Abstract

This paper discusses the advantages of progress testing. A utopia is described where medical schools would work together to develop and administer progress testing. This would lead to a significant reduction of cost, an increase in the quality of measurement and phenomenal feedback to learner and school. Progress testing would also provide more freedom and resources for more creative in-school assessment. It would be an educationally attractive alternative for the creation of cognitive licensing exams. A utopia is always far away in the future, but by formulating a vision for that future we may engage in discussions on how to get there.

## Progress testing

Innovations in education move slowly. An innovative way of assessing knowledge through progress testing was introduced in the late 1970 s [1]. A progress test is a comprehensive written test using preferably scenario-based multiple choice items that is periodically administered to all the students in a curriculum. Naturally with every test administration a new test is developed or new items are retrieved and reviewed from an item bank. Progress testing has many advantages [2, 3], but we restrict ourselves to two. First, progress testing fits with modern constructivist education because it does not reinforce test-directed learning [4] and promotes long-term knowledge and learning methods [5]. Gone are the days when you could train doctors using a finite syllabus. Over a 5-year course the expansion of medical knowledge will be exponential. Repeated testing enhances learning and helps to create the scaffolds for future learning (https://youtu.be/l_x-5haIVRs).

The second advantage is the information that it provides to learners and schools. The learner receives not only profile scores on every test, but also on their longitudinal knowledge growth [6]. Schools can see in which areas the curriculum is effective and which not. When multiple schools participate in progress testing comparative information can be obtained which is even more useful for curriculum evaluation [7]. All depending on the number of tests per year, the number of data points available in a learner’s full curriculum is impressive and provides a wealth of information that no other assessment approach could deliver. There are high correlations between progress tests, allowing early detection of lower performing learners and providing opportunities for early remediation. Despite these advantages, it has taken a long time for schools to adopt this testing approach. In recent years, we see that schools have successfully introduced progress testing [8], also outside medicine [9]. We know of many more schools that have not published about their implementation. In this paper we would like to take a more radical position by using progress testing to significantly advance assessment in medical education. We realize it is utopia, but we would nevertheless make the argument to fuel the discussion about where we should go.

## Progress test utopia

In our utopia schools would work together to collaboratively develop and administer progress testing. It is a waste of resources for schools to reinvent the wheel and start an expensive process of item production and test administration. Progress testing is just that, using tests to measure progress (https://www.youtube.com/watch?v=Mc_-fVmEf1M). That means that individual schools can still have unique curricula and different learning methods. The tests are just the tools to see if the student is making progress within those unique curricula.

In the Netherlands six out of the eight medical schools have combined forces and collaboratively develop and administer progress tests. It has led to an enormous reduction of cost [10], while at the same time it has the benefits as sketched above for the approximately 10,000 learners involved and for the schools themselves. At the same time, it has silenced any discussion on national exams. Naturally, the Netherlands is but a tiny country with few medical schools. Could we not do the same internationally with English speaking schools? Imagine the synergy that this would provide? Let us sketch a few examples of predictable positive developments.

Progress testing with all of its educational benefits would be a low-cost assessment strategy. Schools would have to contribute yearly with a few but high quality items to a central bank. Some cost would be associated with the central administrative and psychometric expertise that is required, but when more schools buy in this would be a marginal cost. Schools would also need to agree on a blueprint for the test. The European Board of Medical Assessors offers progress testing in an online adaptive form (http://www.ebma.eu). This allows a further reduction of the length of the test by about 50%, while the reliability is significantly higher (above 0.90). This is particularly advantageous for students in junior years, a period known to have lower reliabilities in its traditional, paper-based form [2]. With an existing large item bank, schools could flexibly administer progress tests whenever they wish and as frequently as they wish. This would probably deliver the cheapest form of assessment per unit of measurement information in the ability of a test to differentiate learners’ cognitive competence.

Many countries have national licensing exams, but also many countries have no licensing exams. Some countries are preparing or contemplating national exams. Big bang final exams may be useful as a licensing strategy to protect the public [11, 12], but they are not so useful as an educational strategy that would allow both students and schools to improve their performance longitudinally [13]. Moreover, licensing exams are costly. With a comprehensive progress testing approach the need for cognitive national licensing exams evaporates. Written tests predict other written tests and progress tests have shown to accurately predict performance on licencing tests [14]. It is a complete win-win. Schools know how they perform relative to each other, while learners profit from all the feedback. And all of this at a very reasonable cost. With all the knowledge on progress testing, embarking on a traditional licensing exam with all the potential side effects [15] seems almost an outdated strategy.

Finally, an educational synergy should be sketched that might have a phenomenal impact on assessment in medical education. Once a system of progress testing is in place in a school’s assessment strategy, the big question is what it replaces. In some schools the progress test is the only form of knowledge testing [8]. Imagine the resource implications of such a strategy! Other assessment strategies would be possible as well. With a periodic progress test in place, one would have a very precise view of a learner’s knowledge. There is not much point repeating such assessments again locally. It allows assessment in the school to be more creative. With the massive move towards competency-based learning, there is a great need for more authentic forms of assessment and assessing behaviours. It is these assessments that a school might focus on. With the resources being saved, more expenditure is available for these authentic forms of assessment. This would have a massive impact and would drive education in a very desirable direction.

## Conclusion

Utopia is a sketch of the ideal. Given that educational changes move slowly, we are aware we are far away from that ideal. Nevertheless, we might start small and team up with a few medical schools willing to pursue the vision we have shared here: implementing and optimizing a cost-effective assessment tool capable of fostering excellence in learning. It would already be fantastic if some of the current schools using progress testing were to collaborate and reach some of the above synergies. All the psychometric and technological tools necessary for our utopia to become a reality are already available. Together we could make a big difference in assessment in medical education. Let’s start tomorrow.
